# Production and Characterization of Nucleocapsid and RBD Cocktail Antigens of SARS-CoV-2 in *Nicotiana benthamiana* Plant as a Vaccine Candidate against COVID-19

**DOI:** 10.3390/vaccines9111337

**Published:** 2021-11-17

**Authors:** Tarlan Mamedov, Damla Yuksel, Merve Ilgın, Irem Gürbüzaslan, Burcu Gulec, Gulshan Mammadova, Aykut Ozdarendeli, Hazel Yetiskin, Busra Kaplan, Shaikh Terkis Islam Pavel, Muhammet Ali Uygut, Gulnara Hasanova

**Affiliations:** 1 Department of Agricultural Biotechnology, Akdeniz University, Antalya 07058, Turkey; dmlyuksel07@gmail.com (D.Y.); merveilgin.akd@gmail.com (M.I.); irem.gurbuzaslan@gmail.com (I.G.); burcudogusoy@gmail.com (B.G.); gulka2878@gmail.com (G.M.); gulnarahasanova@yahoo.com (G.H.); 2 Department of Microbiology, Medical Faculty, Erciyes University, Kayseri 38280, Turkey; aozdarendeli@erciyes.edu.tr (A.O.); hazelyetiskin@gmail.com (H.Y.); busra.kaplan.3@gmail.com (B.K.); biotech.pavel@outlook.com (S.T.I.P.); mauygut@gmail.com (M.A.U.); 3 Vaccine Research, Development and Application Center, Erciyes University, Kayseri 38280, Turkey

**Keywords:** COVID-19, SARS-CoV-2, nucleocapsid protein, RBD of SARS-CoV-2, plant, transient expression system

## Abstract

The COVID-19 pandemic has put global public health at high risk, rapidly spreading around the world. Although several COVID-19 vaccines are available for mass immunization, the world still urgently needs highly effective, reliable, cost-effective, and safe SARS-CoV-2 coronavirus vaccines, as well as antiviral and therapeutic drugs, to control the COVID-19 pandemic given the emerging variant strains of the virus. Recently, we successfully produced receptor-binding domain (RBD) variants in the *Nicotiana benthamiana* plant as promising vaccine candidates against COVID-19 and demonstrated that mice immunized with these antigens elicited a high titer of RBD-specific antibodies with potent neutralizing activity against SARS-CoV-2. In this study, we engineered the nucleocapsid (N) protein and co-expressed it with RBD of SARS-CoV-2 in *Nicotiana benthamiana* plant to produce an antigen cocktail. The purification yields were about 22 or 24 mg of pure protein/kg of plant biomass for N or N+RBD antigens, respectively. The purified plant produced N protein was recognized by N protein-specific monoclonal and polyclonal antibodies demonstrating specific reactivity of mAb to plant-produced N protein. In this study, for the first time, we report the co-expression of RBD with N protein to produce a cocktail antigen of SARS-CoV-2, which elicited high-titer antibodies with potent neutralizing activity against SARS-CoV-2. Thus, obtained data support that a plant-produced antigen cocktail, developed in this study, is a promising vaccine candidate against COVID-19.

## 1. Introduction

The global public health risk associated with the novel and highly pathogenic coronavirus, currently designated as SARS-CoV-2, continues to grow. As of 3 September 2021, more than 219,979,049 cases were recorded, with more than 4,557,372 confirmed deaths. Vaccination is the only way to prevent infectious diseases, control a pandemic, and reduce morbidity and mortality. Although a number of vaccines (mRNA, adenovirus vector vaccines, and inactivated vaccines) have been approved (22 approved vaccines, see: https://covid19.trackvaccines.org/vaccines/approved/#vaccine-list accessed on 16 September 2021) and are currently used for mass vaccinations, the effectiveness of these vaccines is diminishing due to the emergence of new and recent strains, especially the Delta strain (B.1.617), which has been found in many countries and continues to spread rapidly around the world. Therefore, the world urgently needs more effective COVID-19 vaccines with a stable immune response and long-term immunity against possible emerging mutated variants of SARS-CoV-2 viruses. Since the S protein of SARS-CoV, MERS-CoV, and SARS-CoV-2 contains neutralizing epitopes, it has been shown to be a leading candidate for vaccine developments. In this regard, a number of studies demonstrated that the S protein induced potent humoral and cellular immune responses in tested animal models [1,2,3,4,5]. Most COVID-19 vaccines, which are currently approved or under development (mRNA, DNA, viral vector-based, subunits, and protein-based vaccine) were designed and developed on the basis of the S protein [4,5,6,7,8,9,10,11]. The genome size of SARS-CoV-2 is ~30 kb, one-third of which encodes the S (spike), N (nucleocapsid), E (envelop), and M (matrix) structural proteins. One of the observed features of this virus is that SARS-CoV-2 undergoes about two mutations per month in the global population [12,13]. The emergence of more aggressive mutated strains of the coronavirus has raised concerns regarding the development of more effective COVID-19 vaccines with broader protection. Studies have shown that a mutation in the S1 domain significantly increases the binding affinity for ACE2 and decreases the affinity for neutralizing antibodies [14,15,16,17], which can be logically explained by the high transmissibility and virulence of new strains, including Delta [18,19]. Another structural protein, the N protein, is an important protein involved in RNA binding and required for RNA activity, such as replication. The N protein has been shown to be more conserved compared to the S protein, and it shares 91% similarity with SARS-CoV, in addition to greater stability with fewer observed mutations [20,21,22,23]. Since the N protein has been found to be more conserved and show strong immunogenicity, the N protein could be a strong potential candidate for a COVID-19 vaccine [24,25]. In previous efforts, it was shown that the recombinant N protein produced in *Nicotiana tabacum* significantly induced a humoral immune response [26]. In our pre-published report in BioRxiv [2], for the first time, we described the co-expression of N protein with RBD in *N. benthamiana* plant. Notably, among a number of expression systems, transient expression in plants is a promising platform for the production of recombinant proteins. This system allows the cost-effective production of a variety of complex recombinant proteins, including vaccine candidates, therapeutic proteins, enzymes, and antibodies, in a short period of time, with high yield and fully functional activity [4,27,28,29,30,31,32]. In this study, we report the expression and production of N protein and its co-expression with RBD in *N. benthamiana* plant to produce cocktail antigens of SARS-CoV-2 as a vaccine candidate, which may have broader-spectrum protection.

## 2. Materials and Methods

### 2.1. Cloning and Expression of Nucleocapsid (N) in N. benthamiana

The nucleocapsid gene (1–419 aa GenBank accession YP_009724397) and spike gene of SARS-CoV-2 variant RBD (receptor-binding domain containing fragment, RBD, 319–591 aa, GenBank accession MN985325, Flag- or His6-tagged) were optimized for expression in *N. benthamiana* plants and de novo synthesized at Biomatik Corp. (Kitchener, ON, Canada). To transiently express N, RBD, or N+RBD variants in *N. benthamiana* plants, the *Nicotiana tabacum* PR-1a signal peptide (MGFVLFSQLPSFLLVSTLLLFLVISHSCRA) was added to the N-terminus of N and RBD proteins. In addition, the KDEL sequence (the ER retention signal) and the FLAG epitope (the affinity purification tag for N and RBD proteins) were added to the C-terminus. The resulting sequences were inserted into the pEAQ [33] binary expression vectors to obtain pEAQ-N and pEAQ-RBD. These plasmids were then transferred into the *Agrobacterium* AGL1 strain. To express N, RBD, or N+RBDN+RBD variants in *N. benthamiana* plant, AGL1 harboring pEAQ-N and pEAQ-RBD plasmids was infiltrated into *N. benthamiana* plant leaves. To co-express N and RBD proteins, pEAQ-N and pEAQ-RBD plasmids were infiltrated into plant leaves. Plants were harvested at 4 dpi (day after post infiltration). To co-express N and Endo H, AGL1 strain harboring pEAQ-N was co-infiltrated with pGreen-Endo H construct [31].

### 2.2. Expression Screening of N and N+RBD Variants Produced in N. benthamiana Plant by Western Blot Analysis

SDS-PAGE analyses of plant-produced N, RBD, or N+RBD variants were performed on 10% acrylamide gels stained with Coomassie (Gel Code Blue, Pierce Rockford, IL, USA). Western blot analyses were performed after electrophoresis and transfer of the proteins to polyvinylidene fluoride membranes. After transfer, Western blot membranes were blocked with I-Block (Applied Biosystems, Carlsbad, CA, USA), and recombinant proteins N and N+RBD were detected with anti-DYKDDDDK antibody (cat. no. 651503, BioLegend) or human novel coronavirus nucleoprotein (N) (1–419 aa) monoclonal antibody (MBS7135930, MyBioSource, San Diego, CA, USA).

### 2.3. Purification of Plant-Produced N and N+RBD (Cocktail) Proteins Using Anti-DYKDDDDK Affinity Gel

Purification of plant-produced N and N+RBD variants was performed using anti-FLAG affinity chromatography with anti-DYKDDDDK affinity gel (cat. no. 651503, BioLegend) as described previously [22]. For purification, 20 g of frozen leaves, infiltrated with the pEAQ-N-Flag-KDEL or pEAQ-RBD-Flag-KDEL+ pEAQ-N-Flag-KDEL constructs, were ground in 20 mL of PBS buffer (1× PBS, 150 mM NaCI) using a mortar and a pestle. Plant debris was removed by filtration through Miracloth followed by centrifugation at 20,000× *g* for 25 min and then filtered through a 0.45 μm syringe filter (Millipore, Darmstadt, Germany). An anti-FLAG affinity column was prepared according to the manufacturer’s instructions. Sixty milliliters of a clear supernatant were loaded into a 0.5 mL resin column equilibrated with PBS buffer. The column was washed with 10 volumes of PBS buffer. Bound proteins were eluted using 200 mM glycine, 150 mM NaCl, pH 2.2 into tubes containing 2.0 M Tris solution to neutralize. Total protein content was estimated using the BioDrop and then analyzed by SDS-PAGE and Western blot. The purification yield of purified proteins was calculated and quantified on the basis of SDS-PAGE and WB analyses using highly sensitive Gene Tools software (Syngene, a division of Synoptics Ltd., Cambridge, UK) and ImageJ software, as described previously [4,34].

### 2.4. Glycoprotein Detection

The glycan detection analysis in plant-produced N protein purified using an anti-Flag affinity column was performed by Pro-Q Emerald 300 glycoprotein staining as described previously [4,31]. About 250 ng of the plant-produced N protein, along with standard proteins, was separated on a 10% SDS-PAGE gel, and then the glycans were detected in the gel using Pro-Q Emerald.

### 2.5. Gel Filtration

Gel filtration of plant-produced N and N+RBD proteins was performed with ÄKTA start using a 60 cm × 16 mm column (cat. no. 19-5003-01, GE Healthcare, Chicago, IL, USA), packed with Sephacryl^®^ S-200 HR (cat. no. 17-0584-10, GE Healthcare), as described previously [4]. The column was first equilibrated with 50 mM phosphate buffer, pH 7.4, 150 mM sodium chloride, and then 0.25 mg of plant-produced N and N+gRBD proteins, purified using FLAG affinity chromatography, were loaded onto a column. All eluted fractions from the column were combined and concentrated, buffer exchanged against PBS buffer, concentrated using a Millipore 10K MWCO Amicon Ultra 4 concentrator (cat. no.: UFC8010, Millipore), and analyzed by SDS-PAGE and Western blot analyses.

### 2.6. Stability Assessment of Plant-Produced N and N+gRBD Variants

The stability of plant-produced N and N+gRBD variants was examined using a similar procedure as described previously [4,34]. Briefly, protein samples were diluted to 0.5 mg/mL with PBS and were transferred into low-binding polypropylene Eppendorf tubes. After incubation at 37 °C for 48, 96, 72, and 96 h, samples were analyzed by SDS-PAGE. The degradation of N protein bands was calculated and quantified on the basis of SDS-PAGE and WB analyses using highly sensitive Gene Tools software (Syngene Bioimaging, Cambridge, UK) and ImageJ software, as described previously [4,34].

### 2.7. Immunogenicity Studies of N and N+gRBD in Mice

Immunogenicity studies of plant-produced N and N+gRBD proteins were performed in groups of 6–7 week old Balb/c male animals (six mice/group) as described recently [4]. Mice were immunized intramuscularly (IM) on days 0 and 21 with 5 µg of N and N+RBD adsorbed to 0.3% Alhydrogel. Blood samples were taken from immunized mice on days 21 and 42, and antibody levels in blood serum were determined by ELISA. ELISA plates were coated with 100 ng/well N, gRBD, or N+RBD proteins diluted in coating buffer. Mice receiving PBS with Alhydrogel were used as control group. Serum dilutions of 102 to 108 were used to determine IgG titers. In addition to determination of IgG titers, end-point titers corresponding to serum dilutions of sera giving a mean OD value four times greater than the control sample were also calculated. In order to discriminate RBD-specific IgG and N-specific IgG antibodies in the sera of mice vaccinated with RBD + N protein, ELISA plates were coated with 100 ng/well N, gRBD, or N+RBD proteins diluted in coating buffer. Then, 10^2^ to 10^8^ dilutions of the sera of mice vaccinated with RBD + N protein were used for ELISA immunogenicity analyses to determine RBD-specific IgG and N-specific IgG titers.

Mice studies were performed with the permission of the Akdeniz University (Antalya, Turkey) Animal Experiments Local Ethics Committee in Akdeniz University Experimental Animal Care Unit under the supervision of a veterinarian (Protocol no: 1155/2020.07.01) in accordance with animal experiment guidelines and regulations approved by the Ethics Committee and ARRIVE guidelines.

### 2.8. SDS-PAGE and Western Blot Analyses of Purified N and N+gRBD Variants

SDS-PAGE analyses of plant produced N and N+gRBD variants were performed on 10% acrylamide gels stained with Coomassie (Gel Code Blue, Pierce Rockford, IL, USA). Western blot analyses were performed after electrophoresis and transfer of the proteins to polyvinylidene fluoride membranes (PVDF). After transfer, Western blot membranes were blocked with I-Block (Applied Biosystems, Carlsbad, CA, USA), and recombinant proteins were detected with anti-FLAG. The image was taken using a highly sensitive GeneGnome XRQ Chemiluminescence imaging system (Syngene, a Division of Synoptics Ltd., Cambridge, UK). The amount of each protein (N or RBD) in the cocktail antigen (RBD + N) was quantified by SDS-PAGE analysis using BSA as a standard. Protein bands were quantified using highly sensitive Gene Tools software (Syngene Bioimaging, UK) and ImageJ software (https://imagej.nih.gov/ij accessed on 9 November 2020).

### 2.9. Virus Titration

African green monkey kidney clone E6 (Vero E6) cells (ATCC, no. CRL 1586, Manassas, VA, USA) were grown in Dulbecco’s modified Eagle’s medium (DMEM) supplemented with 10% heat-inactivated fetal bovine serum (FBS) and 100 mM L-glutamine (Sigma–Aldrich, Darmstadt, Germany). The hCoV-19/Turkey/ERAGEM-001/2020 strain was used for the titration assay [35]. The virus titer was determined using the tissue culture infective dose 50% (TCID_50_) method. Briefly, Vero E6 cells (0.4 × 10^6^ cells/mL) were seeded in 96-well plates and incubated for 18–24 h at 37 °C. The virus was serially diluted 10-fold in DMEM and added to 96-well culture plates and cultured for 5–7 days in a 5% CO_2_ incubator at 37 °C, and cells were examined for cytopathic effect (CPE) under a microscope. The TCID_50_ was calculated using the Reed and Muench method. All experiments with infectious SARS-CoV-2 were performed in a biosafety level 3 (BSL3) enhanced facility at Erciyes University Vaccine Research, Development and Application Center (ERAGEM).

### 2.10. Microneutralization Test (MNT)

SARS-CoV-2–specific neutralizing antibody titers were measured using a microneutralization test (MNT) as described previously [4]. Briefly, serum samples were heat-inactivated at 56 °C for 1 h prior to use. Serum dilutions were 1/8–1/512 in twofold increments and were incubated with the 100 TCID_50_ of virus for 1 h at 37 °C. The mixtures were then added to 96-well plates containing confluent Vero E6 cell monolayers. The inoculum was decanted, and DMEM with 2% FBS was added. The microplates were formatted to test three wells for each serum dilution, six virus-only control wells, and three blank wells containing growth medium alone. Cells were assessed daily for CPE and listed at 3–4 days post infection (dpi). The 50% neutralization titer (NT_50_) was calculated as the highest dilution of the serum at which the infectivity was neutralized in 50% of the cell in wells. Seropositivity was defined as a titer ≥1/8.

### 2.11. Statistical Analysis

All statistical analyses were performed using a GraphPad Prism software. One-way ANOVA was used for all experiments. One-way ANOVA was used to compare antibody responses of sera immunized by N or N+RBD and to test the neutralization ability of the sera from mice immunized with the plant-produced N and N+RBD proteins against live SARS-CoV-2 infection. Results were considered statistically significant at *p* < 0.05, and *p*-values were denoted as * *p* < 0.05, ** *p* < 0.01, and *** *p* < 0.001. Each point on the graphs was derived from three replicates for each dilution.

## 3. Results

### 3.1. Engineering, Cloning and Expression, Purification, and Characterization of N Protein in N. benthamiana Plant

**Figure 1:**
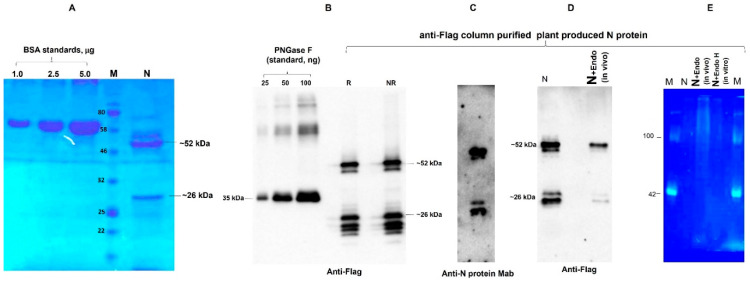
SDS-PAGE (**A**), Western blot (**B**–**D**), and glycan detection (**E**) analysis of purified plant-produced N protein. N protein from *N. benthamiana* plant was purified using anti-FLAG antibody resin. Membranes were probed with anti-Flag (**B**,**D**) or anti-N protein monoclonal antibody (human novel coronavirus nucleoprotein, 1–419 aa, monoclonal antibody, (**C**)). (**A**) Anti-FLAG antibody resin-purified N protein was analyzed by SDS-PAGE. N: 2 μg of plant-produced, purified N protein was loaded into a well. BSA standards: 1.0, 2.5, and 5.0 μg of BSA protein was loaded as a standard protein. M: color pre-stained protein standard (NEB, Ipswich, MA, USA). (**B**) Plant-produced N protein was run in reducing (R) or nonreducing condition (NR). Lanes: 25, 50, and 100 ng of plant-produced PNGase was loaded as a standard protein. (**C**) Anti-Flag column-purified N protein was analyzed by Western blot analysis using anti-N protein mAb. (**E**) Glycan detection analysis of anti-Flag purified N protein (N) in vivo co-expressed with Endo H (N + Endo H, in vivo). M—CandyCane glycoprotein molecular weight standards (Molecular Probes), 250 ng of each protein per lane. (**D**)) Western blot analysis of the same sample using the anti-FLAG antibody. (Figure 1A was cropped from Figure S1, Figure 1B was cropped from Figure S2, Figure 1C was cropped from Figure S3, Figure 1D was cropped from Figure S4 and Figure 1E was cropped from Figure S5).

The engineering, cloning, and expression of N protein were performed as described in Section 2. The expression level of N protein determined by Western blot analysis and ELISA was about 45 mg/kg of plant leaves. The plant-produced N protein was purified using single-step anti-FLAG affinity chromatography. The purification yield was about 22 mg pure protein/kg of plant biomass for Flag-tagged N protein. On SDS-PAGE and Western blotting, the plant-produced N protein molecule appeared as a doublet protein with an MM of ~48 and ~24 kDa (Figure 1A,B). Both molecular forms of plant-produced N proteins were recognized by anti-FLAG antibody (Figure 1B) and by N protein-specific mAb (Figure 1C), demonstrating specific reactivity of mAb to the two forms; it also confirmed that epitopes for mAb are still present in the ~24 kDa fragment. Figure 1B demonstrates the SDS-PAGE followed by Western blotting of plant-produced N protein, performed under reducing and nonreducing conditions. The N protein of SARS-CoV-2 has five potential *N*-glycosylation sites. Therefore, we co-expressed the N protein gene with bacterial Endo H genes to confirm the *N*-glycosylation status of plant-produced N protein. When the N protein was co-expressed with Endo H, there was no protein band shift in the Western blotting (Figure 1D). In addition, we performed glycan detection analysis of plant-produced N protein; no glycan was detected in plant-produced N protein (Figure 1E), suggesting that plant-produced N protein is not *N*-glycosylated.

### 3.2. Co-Expression of N and gRBD, and Production and Purification of N+gRBD Antigens from N. benthamiana Plant

**Figure 2:**
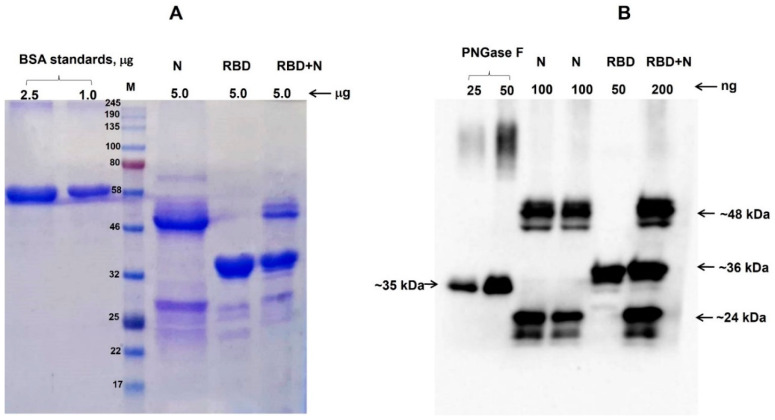
SDS-PAGE (**A**) and Western blot (**B**) analysis of co-expression of RBD with N protein. (**A**) SDS-PAGE analysis of plant-produced, anti-Flag affinity column-purified N, RBD, and N+RBD proteins. N: plant-produced purified N protein; RBD: plant-produced purified RBD protein [4]; RBD + N: RBD + N protein, purified from *N. benthamiana* plant, co-expressed with N and RBD constructs. BSA standards: 1.0 and 2.5 μg of BSA protein was loaded as a standard protein. M: color pre-stained protein standard (NEB, Ipswich, MA, USA). (**B**) Western blot analysis of plant-produced, anti-Flag affinity column-purified N, RBD, and N+RBD proteins. The proteins were loaded into wells as indicated; PNGase F: 25 or 50 ng of plant-produced PNGase F was used as a control protein. (Figure 2A was cropped from Figure S6 and Figure 2B was cropped from Figure S7).

Co-expression of N protein with RBD was performed as described in Section 2. To produce antigen cocktails containing N+RBD proteins, agrobacterium harboring pEAQ-N and pEAQ-RBD constructs was infiltrated into plants, and plant leaves were harvested at 4 dpi (day of post infiltration). N protein and N+gRBD co-expressed (cocktail) protein were purified using single-step anti-FLAG affinity chromatography. SDS-PAGE (Figure 2A) and Western blot (Figure 2B) analysis of the cocktail protein purified from *N. benthamiana* plant demonstrated three major proteins with MM 48 kDa (N protein), 36 kDa (gRBD), and 24 kDa (N protein) in the cocktail antigens.

### 3.3. Gel Filtration Chromatography of N and N+RBD Proteins

**Figure 3:**
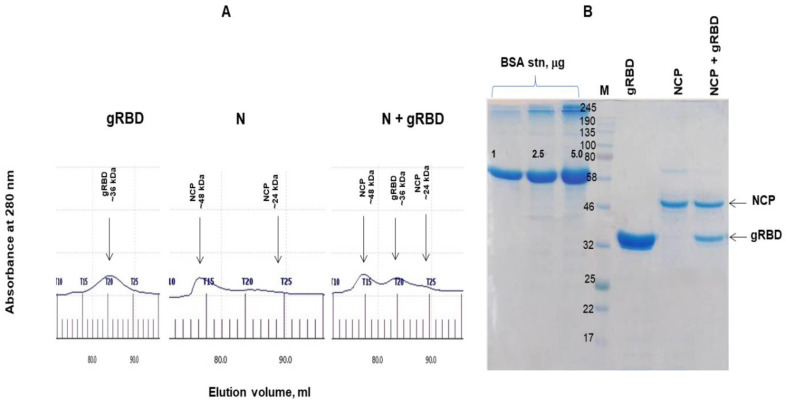
Gel filtration chromatography and SDS-PAGE analysis of RBD, N, and N+RBD proteins, eluted from a Sephacryl^®^ S-200 HR column. (**A**) Profiles of plant-produced gRBD [4], N, and N+gRBD proteins, eluted from a Sephacryl^®^ S-200 HR column. The column was equilibrated with 50 mM phosphate buffer, pH 7.4, containing 150 mM NaCl. The plant-produced, FLAG affinity chromatography-purified gRBD, N, and N+gRBD proteins (0.25 mg) were loaded onto the column. Gel filtration was performed with ÄKTA start using a 60 cm × 16 mm column (cat. no. 19-5003-01, GE Healthcare, Chicago, IL, USA), packed with Sephacryl^®^ S-200 HR (cat. no. 17-0584-10, GE Healthcare). (**B**) SDS-PAGE analysis of plant-produced gRBD [4], N, and N+gRBD proteins eluted from Sephacryl^®^ S-200 HR column. gRBD: plant-produced RBD (~36 kDa) [4]; N: plant-produced N protein (~48 kDa); N+gRBD: antigen cocktail containing N protein (~48 kDa) and gRBD (~36 kDa); BSA stn: 1.0, 2.5, and 5.0 μg of BSA protein was loaded as a standard protein. M: color pre-stained protein standard (NEB, UK). (Figure 3B was cropped from Figure S8).

Anti-Flag affinity chromatography-purified N and N+RBD proteins were further analyzed by gel filtration through a Sephacryl S-200 column (Figure 3A). The N protein was eluted from the S-200 column as double peaks, and no dimerization or aggregation was observed. Similarly, N+gRBD proteins were eluted from the S-200 column as triple peaks, and no dimerization or aggregation was observed. Figure 3B demonstrates the SDS-PAGE analysis of a full-length (~48 kDa) N protein, with antigen cocktails containing the ~48 kDa N protein and 36 kDa gRBD protein, eluted from the Sephacryl S-200 column and concentrated.

### 3.4. Stability Assessment of N Protein

**Figure 4:**
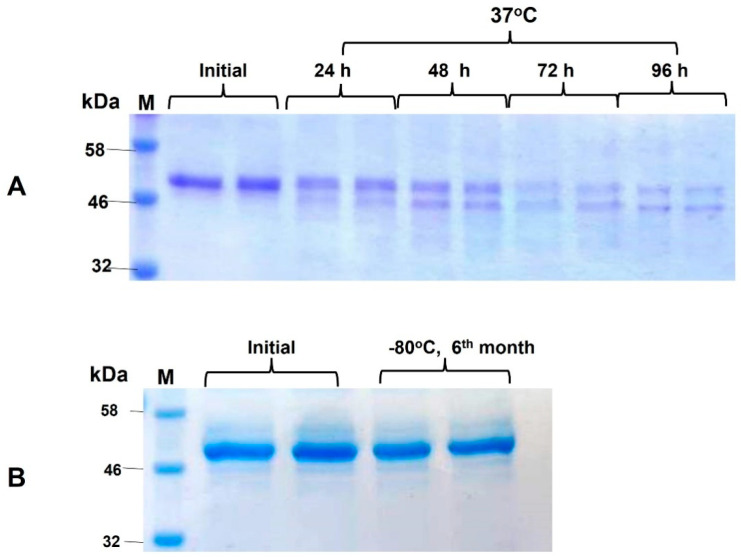
Stability of plant produced N protein. (**A**) Plant-produced, FLAG antibody affinity column-purified N protein was incubated at 37 °C for 24, 48, 72, and 96 h, and analyzed in SDS-PAGE. Lanes were loaded with ~2.0 μg of N protein. (**B**) Plant-produced, FLAG antibody affinity columnpurified N protein was stored at −80 °C for 6 months and then analyzed in SDS-PAGE M: color pre-stained protein standard. (Figure 4A was cropped from Figure S9 and Figure 4B was cropped from Figure S10).

The stability analysis of plant-produced RBD was recently reported [4]. The stability analysis of plant-produced N protein (~48 kDa, full length) was examined after incubation at 37 °C for 24, 48, 96, 72, and 96 h. The procedure of stability analysis was similar to that described previously [32]. These analyses showed that the N protein was less than 50% degraded after incubation at 37 °C for 48 h (Figure 4A). There was almost no degradation of plant-produced N protein during a 6 month period when stored at −80 °C (Figure 4B).

### 3.5. Immunogenicity Studies of N and N+RBD in Mice

**Figure 5:**
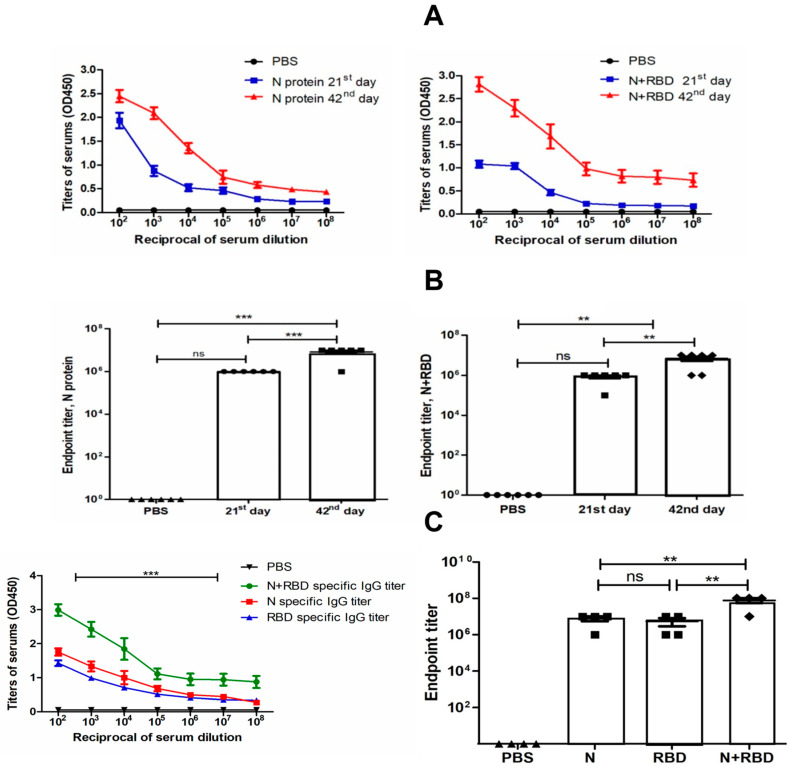
Immunogenicity of plant-produced N protein and N+RBD in mice. First, 6–7 week old Balb/c male mice were immunized on IM with 5 μg of plant-produced N and N+RBD using Alhydrogel as an adjuvant on the 0th and 21st days. IgG titers of mice immunized with plant-produced N and N+RBD antigens were determined by ELISA from sera collected on the 21st and 42nd days after injection. For the detection of IgG titers, mouse sera on the 21st day and 42nd days were evaluated by 10^2^ to 10^8^ serum dilutions. (**A**) Detection of specific IgG titers in 21st and 42nd day sera from mice immunized with N and N+RBD. (**B**) Detection of endpoint titers in sera with different dilutions specific to N and N+RBD on days 21 and 42. (**C**) Determination of specific IgG titer for N, RBD, and N+RBD antigens in mice immunized with plant-produced N+RBD. First, 6–7 week old Balb/c male mice were immunized on IM with 5 μg of plant-produced RBD [4] and N+RBD using Alhydrogel as an adjuvant on the 0th and 21st days. Specific IgG titers of mice immunized with plant-produced N+RBD were determined by ELISA from sera collected on the 42nd day after injection. The ELISA plate was coated with 100 ng of plant-produced N, RBD [4], and N+RBD proteins. (**C**) (left) N-, RBD- [4], and N+RBD-specific IgG titers on 42nd day sera from mice immunized with N+RBD. (**C**) (right) Detection of endpoint titers in mice sera immunized with N+RBD with different dilutions specific to N, RBD, and N+RBD on day 42. Endpoint titer was derived from reciprocal dilutions of sera, giving an OD value four times greater than mouse sera receiving PBS with Alhydrogel as a control sample. Data in the graph are shown as the mean ± standard error of the mean (SEM) of triplicates at each sample dilution. Statistical analysis was performed using Tukey’s multiple comparison tests; ** *p* < 0.01, and *** *p* < 0.001 (*n* = 6 mice/group).

Antibody levels were determined by ELISA on 21st and 42nd day mouse sera immunized with two doses of N and N+RBD. As shown in Figure 5A,B, the plant-produced N and N+RBD proteins were able to induce significantly high titers of antibody with a 5 µg dose. On the other hand, the endpoint titer of N+RBD obtained by co-expression of gRBD with N protein was 10^7^ in this study. The endpoint titer of gRBD was demonstrated to be 10^6^ in a recently published study [4]. These immunogenicity analysis results demonstrate that N+RBD elicits high-titer antibodies compared to RBD or N proteins.

### 3.6. Neutralization Activity Assessment of N or N+RBD by Microneutralization Test

**Figure 6:**
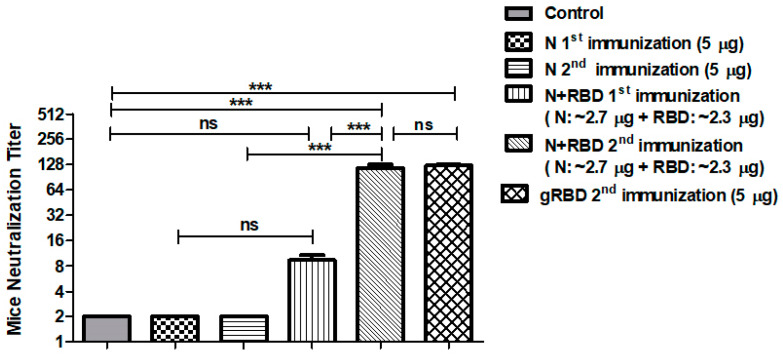
In vitro microneutralization assay of mouse sera against live SARS-CoV-2 in the Vero E6 cell line immunized with plant-produced RBD [4], N, or N+RBD proteins. In vitro microneutralization assay of 21st and 42nd day mouse sera immunized with plant-produced RBD [4] and N+RBD (~2.7 μg + 2.3 μg, respectively) against live SARS-CoV-2 in the Vero E6 cell line as indicated. gRBD 2nd immunization (5 μg): in vitro microneutralization of sera from mice immunized with 5 μg of gRBD on day 42 [4]. The experiment was performed using 32 to 1024 dilutions of mouse sera collected on day 21 and day 42. One-way ANOVA Tukey’s multiple comparison tests were used to calculate statistical significance (*n* = 6 mice/group); *** *p* < 0.001.

We further tested the anti-SARS-CoV-2 neutralization activity of sera from mice immunized with the plant-produced, anti-FLAG affinity chromatography-purified N and N+gRBD proteins against live SARS-CoV-2 infection in Vero E6 cells. The results are presented in Figure 6. Sera collected from mice immunized with 5 μg of plant-produced N protein (adjuvanted with Alhydrogel) collected on day 21 (after the first immunization) or 42 (after the second immunization) showed no neutralizing ability. Mice sera collected from mice immunized with 5 μg of plant-produced N+gRBD (adjuvanted with Alhydrogel) collected on day 21 (after the first immunization) or 42 (after the second immunization) displayed SARS-CoV-2 neutralization activity at 1:8 and 1:128, respectively. Murine sera, collected from mice immunized with 5.0 μg of plant-produced gRBD (alone), collected on day 42 (after the second immunization) displayed SARS-CoV-2 neutralization activity at 1:128, which is consistent with the results we recently reported [4]. Similar neutralization activity (at 1:128) was observed with the sera of mice immunized with ~2.3 μg of gRBD but in combination with ~2.7 μg of N protein (~2.3 μg of gRBD + ~2.7 μg of N protein (total 5.0 μg) in immunized mice), demonstrating that the N protein possibly contributes to the enhancement of neutralizing activity.

## 4. Discussion

SARS-CoV-2 is a novel and highly pathogenic coronavirus that began an outbreak in Wuhan, China in 2019 and continues to spread rapidly around the world. A new strain of SARS-CoV-2, Delta, has emerged that is more aggressive than previous variants with greater transmission and spread. Currently, several types of vaccines such as mRNA, DNA, viral vector-based, subunits, and protein-based types have been developed, approved, and used for mass immunization of the world’s population. The US Food and Drug Administration approved the first COVID-19 vaccine known as the Pfizer-BioNTech COVID-19 vaccine. However, the effectiveness of these vaccines is significantly reduced in relation to newer strains, especially Delta (B.1.617). Since mutations affect the S protein–ACE2 receptor interaction, they could potentially affect the effectiveness of vaccines and drugs, which were designed on the basis of the receptor-binding motif (RBM). It should be noted that SARS-CoV-2 has been shown to undergo about one or two mutations per month [12,13]. A SARS-CoV-2 spike protein amino-acid change, D614G, is dominant in most places around the globe [36]. D614G viruses exhibit increased sensitivity to neutralizing antibodies, likely due to the effect of the mutation on the molecular dynamics of the S protein [37,38,39]. Recent studies demonstrated that SARC-Cov-2 infectivity, human-to-human transmission, and immune escape are significantly influenced by RBM mutations [40]. Currently, multiple new variants of concern have emerged and are circulating globally, such as the Alpha (known as B.1.1.7, British variant), Beta (B.1.351, South African), Gamma (first reported in Brazil), and Delta (B.1.617.2, Indian variant), which are associated with enhanced transmissibility and increased virulence. The British B.1.1.7 variant has a total of 17 mutations, eight of which are in the spike protein, including N501Y, A570D, P681H, T716I, S982A, and D1118H, in addition to Δ69–70 and Δ144 deletions. All four reported variants (Alpha, Beta, Gamma, and Delta) have mutations in the RBD and the NTD, and the N501Y mutation is common to all variants except the Delta. The N501Y mutation results in increased affinity of the spike protein to ACE2 receptors, thereby enhancing the viral attachment and entry into host cells [41,42,43]. The Beta (B.1.351, South African) variant has a total of nine mutations (L18F, D80A, D215G, R246I, K417N, E484K, N501Y, D614G, and A701V) in the spike protein, and three mutations (K417N, E484K, and N501Y) are located in the RBD. Mutations in the RBD have been shown to result in increased binding affinity for the ACE2 receptor [43,44]. The Delta (B.1.617.2) variant has a total of 10 mutations (T19R, G142D, R158G, L452R, T478K, D614G, P681R, and D950N, in addition to two deletions, Δ156 and Δ157) in the spike protein, including two deletions, five mutations in the NTD, two mutations (L452R, T478K) in the RBD, one mutation close to the furin cleavage site (P681R), and one in the S2 region (D950N) [45]. Reduced sensitivity of this SARS-CoV-2 variant Delta to antibody neutralization has been observed; this variant was resistant to neutralization by some anti-NTD (N-terminal domain) and anti-RBD monoclonal antibodies [45]. In addition to efficacy and quality, the development of safer vaccines and pharmaceutical products is critical to successful vaccination. Recent studies have shown that the frequency of allergic reactions to currently used COVID-19 vaccines is higher than that observed for other vaccines [46] despite the fact that most of the available COVID-19 vaccines do not contain potentially sensitizing compounds [46]. Anaphylaxis can also be caused by the production of high concentrations of IgG antibodies that bind to the Fc gamma receptor, present on the surface of certain cells such as macrophages, neutrophils, eosinophils, basophils, human platelets, mast cells, B lymphocytes, follicular dendritic cells, and natural killer cells [47]. This is another advantage of recombinant protein-based vaccines, whereby the IgG concentration can be controlled by controlling the dosage of the vaccine. Notably, subunit vaccines based on recombinant proteins have been shown to be safer and cause fewer side-effects than other vaccine types, especially inactivated and live-attenuated vaccines [48]. Thus, the world still urgently needs to develop more effective and safer alternative SARS-CoV-2 vaccines, as well as antiviral and therapeutic drugs, to prevent the disease and stop the SARS-CoV-2 pandemic. SARS-CoV-2 is a single-stranded RNA virus; one-third of its genome (~30 kb) encodes S, N, E, and M structural proteins. The S protein has two subunits, S1 and S2, and plays a key role in virus binding, fusion, and entry into host cells. The S1 domain of S glycoprotein contains an RBD and plays a key role in the specific binding to its receptor, ACE2. Notably, a number of studies have shown the S protein as a leading target for the development of SARS-CoV-2 vaccines; therefore, most of the COVID-19 vaccines currently developed and available are S protein-, specifically RBD-based vaccines [3,4,8,9,10,11]. The RBD, which is a critical region for receptor binding, has been selected by various research groups as the main target for the development of a SARS-CoV-2 vaccine [4,5,49,50]. We recently reported an RBD-based SARS-CoV-2 vaccine produced by the *N. benthamiana* plant [4]. We demonstrated that, in mice, the plant-produced gRBD and dRBD antigens elicited high titers of antibodies with a potent virus-neutralizing activity [4]. We showed that the selection of amino-acid regions of RBDs is crucial for high-yield production of a functionally active and soluble protein [4]. In this study, we developed N protein and an antigen cocktail comprising RBD and N proteins of SARS-CoV-2 as vaccine candidates against SARS-CoV-2 infection. The antigen cocktail was produced by co-expression of the RBD of S [4] and N proteins of SARS-CoV-2 in *N. benthamiana* plant. Although plant-produced N protein did not induce neutralizing responses in mice, our hypothesis is that an antigen cocktail may generate a durable immune response and additional protective effects compared to RBD alone. In addition, N protein has been shown to be highly immunogenic and, most importantly, more conserved among SARS-CoV-2 variants and other coronaviruses [24]. In fact, fewer mutations have been observed in the N protein over time [20,23,51,52,53]. It has also been recently shown that the crystal structure of the SARS-CoV-2 nucleocapsid protein [54] is very similar to coronavirus N proteins, which were previously described. Cong et al. (2020) using a mouse hepatitis virus model showed that the nucleocapsid protein contributes to forming helical ribonucleoproteins during the packaging of the RNA genome, thereby regulating viral RNA synthesis during replication and transcription [55]. A number of studies have shown the critical roles of N protein at multiple stages of the viral lifecycle [24]. It was also demonstrated that N proteins of many coronaviruses are highly immunogenic and are produced abundantly during infection [53], and high levels of IgG antibodies against N protein have been detected in sera from patients who recovered from SARS [56]. In this study, we also show that plant-produced N and N+RBD antigens were able to induce significantly high titers of antibodies with Alhydrogel adjuvant with potent virus-neutralizing activity; the cocktail antigen elicited high-titer antibodies compared to RBD or N proteins. Thus, an N+gRBD antigen cocktail is a promising vaccine candidate against COVID-19.

## 5. Conclusions

As SARS-CoV-2 mutations appear worldwide, the effectiveness of existing vaccines against variants is declining. The N-protein + RBD multi-antigen vaccine approach that we proposed, designed, and produced for the first time [2], which was widely discussed in a recently published review article [24], could be a promising approach for the production of effective vaccines against emerging SARS-CoV-2 variants, given that the N-protein is highly immunogenic, more conserved, and less vulnerable to possible emerging mutations. The multi-antigen vaccine approach can be applied to existing COVID-19 vaccines, in particular mRNA, DNA, viral vectors, and other types of vaccines, to be effective toward new emerging variants. Plant-produced N or N+RBD antigens can also be used as a diagnostic reagent in serological tests for the detection of SARS-COV-2 antibody in COVID-19 patients.

## Data Availability

Not applicable.

## Supplementary Materials

The following are available online at https://www.mdpi.com/article/10.3390/vaccines9111337/s1, Figure S1: SDS-PAGE analysis of purified plant-produced N protein. Figure 1A was cropped from this SDS-PAGE gel image; Figure S2: Western blot analysis of purified plant-produced N protein. Plant-produced N protein was run in reducing (R) or nonreducing condition (NR). Figure 1B was cropped from this western blot image; Figure S3: Anti-Flag column-purified N protein was analyzed by Western blot analysis using anti-N protein mAb. Figure 1C was cropped from this western blot image; Figure S4: Western blot analysis of anti-Flag purified N protein (N) in vivo co-expressed with Endo H (N+Endo H) in vivo. Figure 1D was cropped from this western blot image; Figure S5: Glycan detection analysis of purified plant-produced N protein. Figure 1E was cropped from this Glycan detection image; Figure S6: SDS-PAGE analysis of co-expression of RBD with N protein. Figure 2A was cropped from this SDS-PAGE gel mage; Figure S7: Western blot analysis of co-expression of RBD with N protein. Figure 2B was cropped from this western blot mage; Figure S8: SDS-PAGE analysis of RBD, N, and N+RBD proteins, eluted from a Sephacryl^®^ S-200 HR column. Figure 3B was cropped from this SDS-PAGE gel image; Figure S9: Stability of plant-produced N protein. Plant-produced, FLAG antibody affinity column-purified N protein was incubated at 37 °C for 24, 48, 72, and 96 h, and analyzed in SDS-PAGE. Figure 4A was cropped from this SDS-PAGE gel mage; Figure S10: Plant-produced, FLAG antibody affinity column-purified N protein was stored at −80 °C for 6 months and then analyzed in SDS-PAGE M: color pre-stained protein standard. Figure 4B cropped from this SDS-PAGE gel mage.
