# Supplementary material for: Production and Characterization of Nucleocapsid and RBD Cocktail Antigens of SARS-CoV-2 in Nicotiana benthamiana Plant as a Vaccine Candidate against COVID-19
Source: Vaccines (Basel). 2021 Nov 17;9(11):1337. doi: 10.3390/vaccines9111337 (PMC8621474; doi:10.3390/vaccines9111337)
Supplement: Supplementary file 1 [file vaccines-09-01337-s001.zip › vaccines-1396972-supplementary.pdf]

Full-length gels and blots

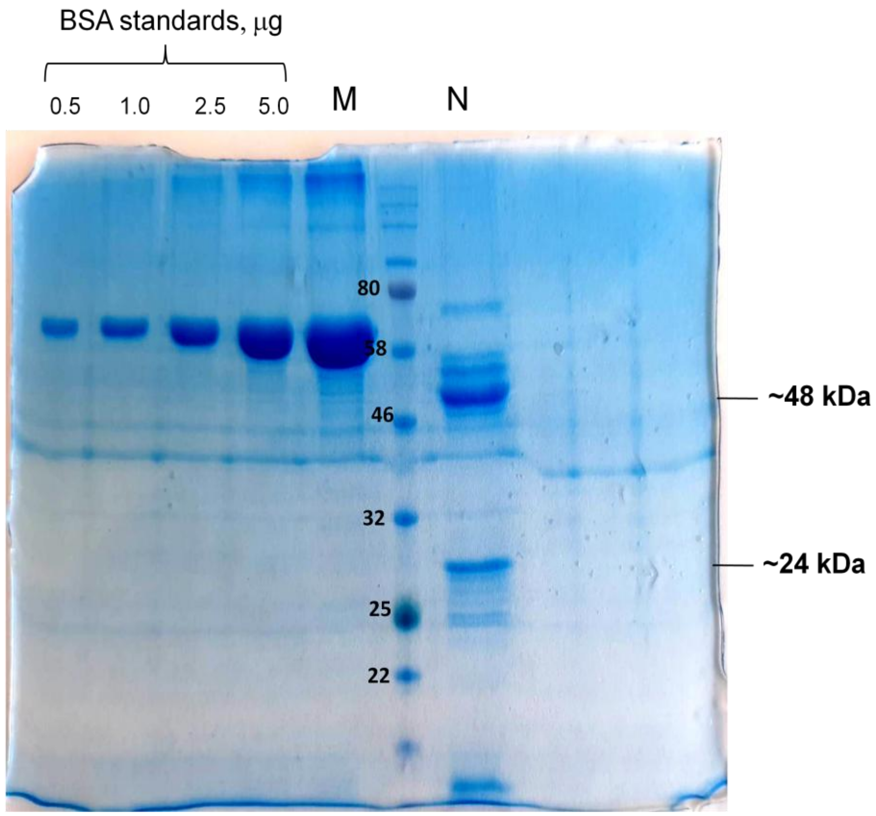

Supplementary Figure S1. SDS-PAGE analysis of purified plant-produced N protein. Figure 1A was cropped from this SDS-PAGE gel image.

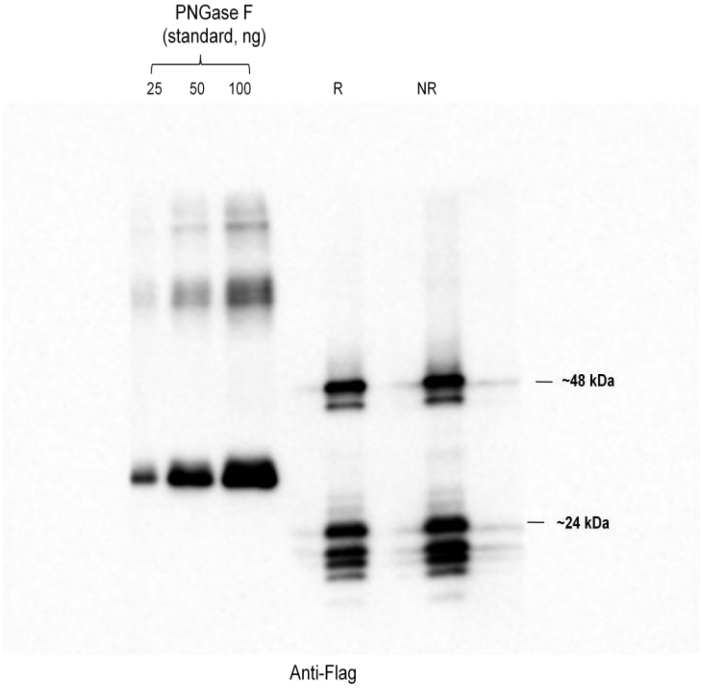

Supplementary Figure S2. Western analysis of purified plant-produced N protein. Plant-produced N protein was run in reducing (R) or nonreducing condition (NR). Figure 1B was cropped from this western blot image.

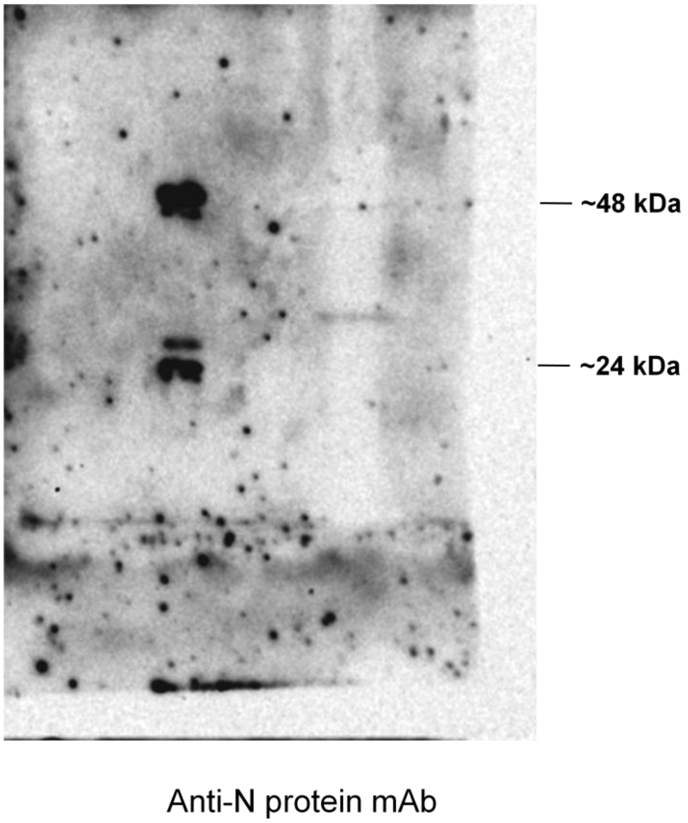

Supplementary Figure S3. Anti-Flag column-purified N protein was analyzed by Western blot analysis using anti-N protein mAb. Figure 1C was cropped from this western blot image.

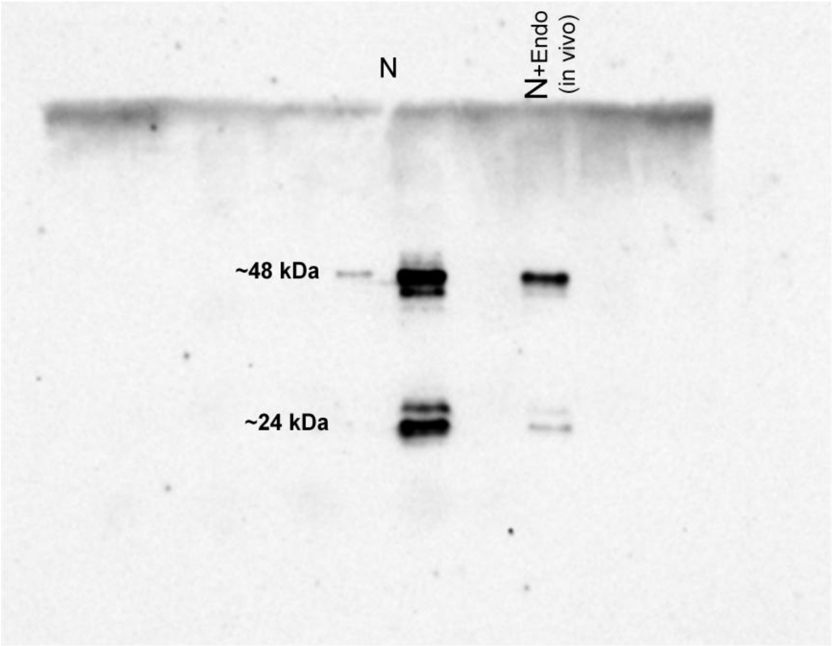

Anti-Flag

Supplementary Figure S4. Western blot analysis of anti-Flag purified N protein (N) in vivo co-expressed with Endo H (N+ Endo H) in vivo. Figure 1D was cropped from this western blot image.

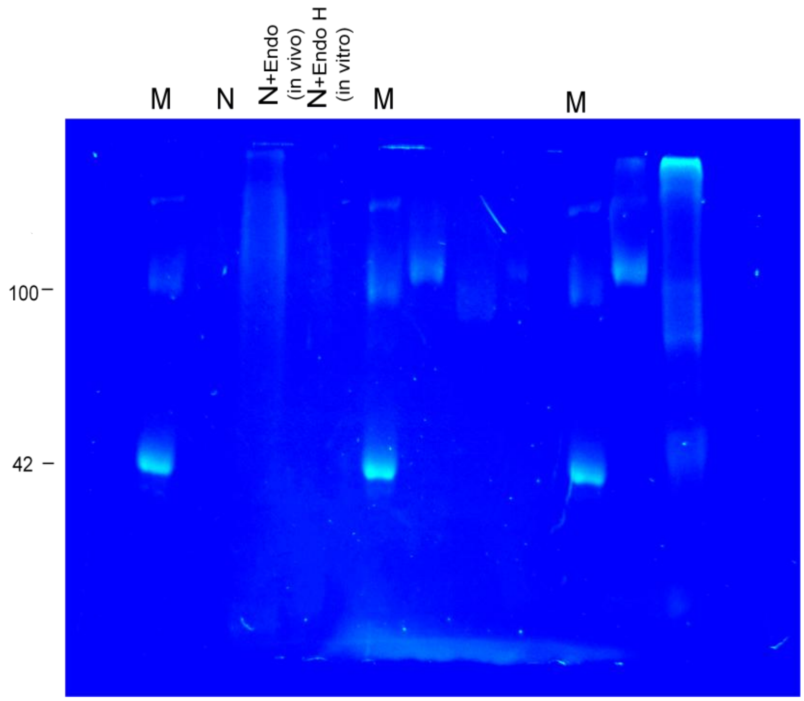

Supplementary Figure S5. Glycan detection analysis of purified plant-produced N protein. Figure 1E was cropped from this Glycan detection image.

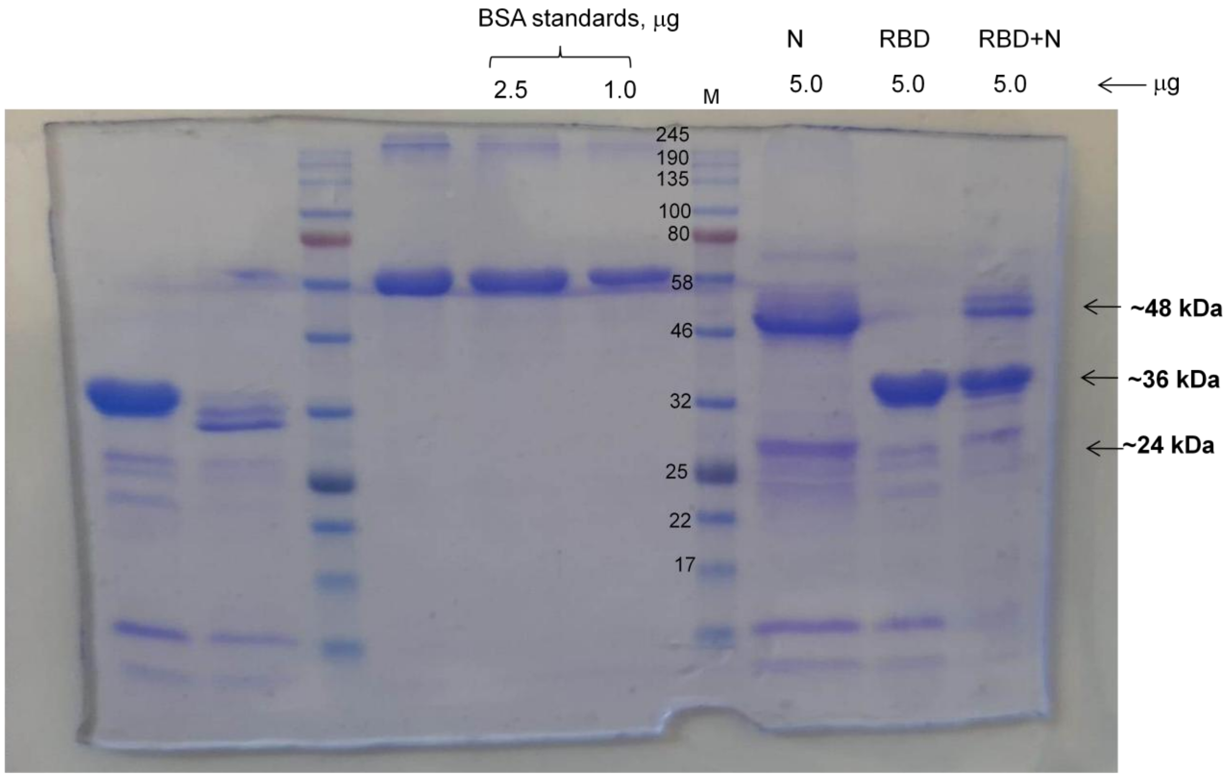

Supplementary Figure S6. SDS-PAGE analysis of co-expression of RBD with N protein. Figure 2A was cropped from this SDS-PAGE gel mage.

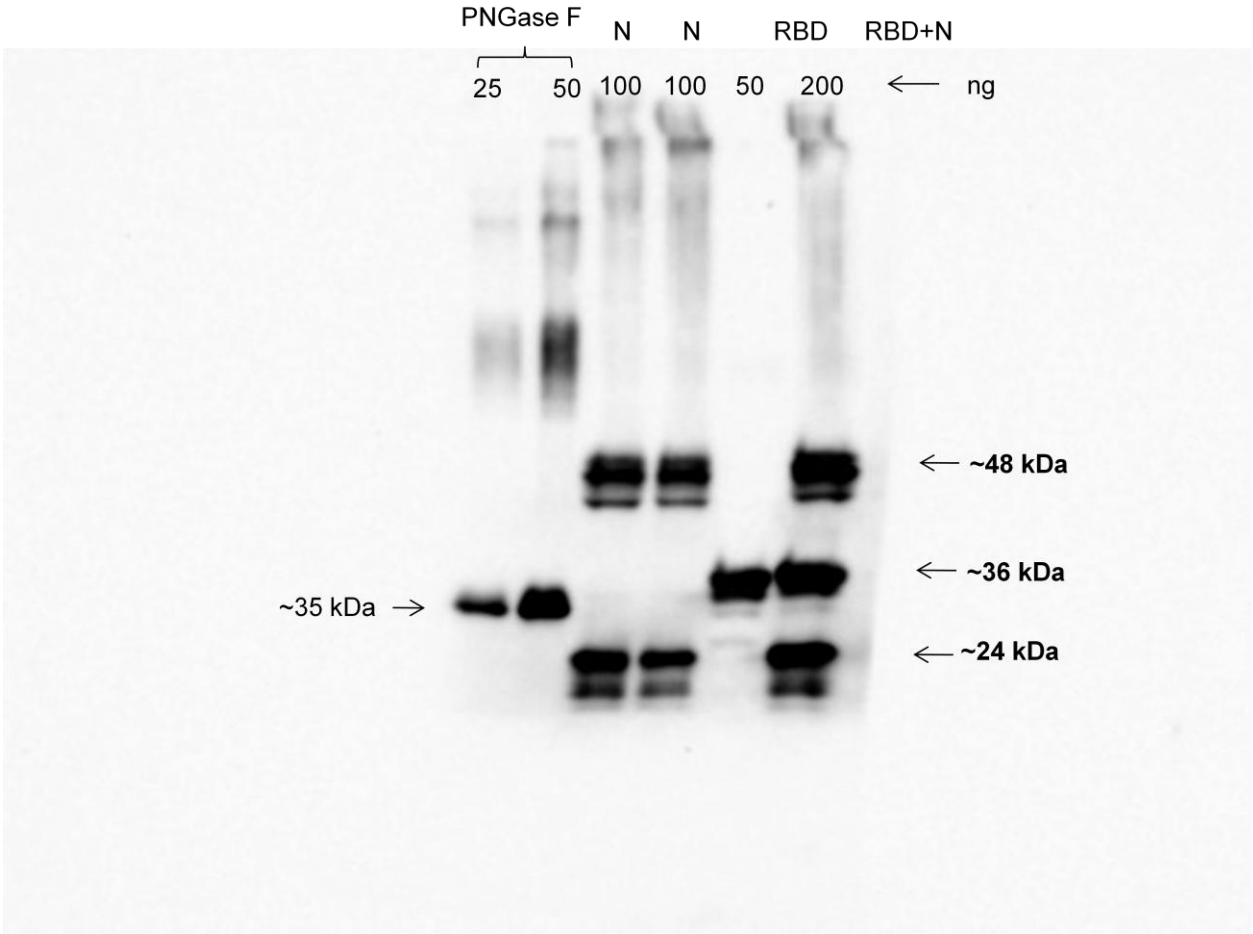

Supplementary Figure S7. Western blot analysis of co-expression of RBD with N protein
Figure 2B was cropped from this western blot mage.

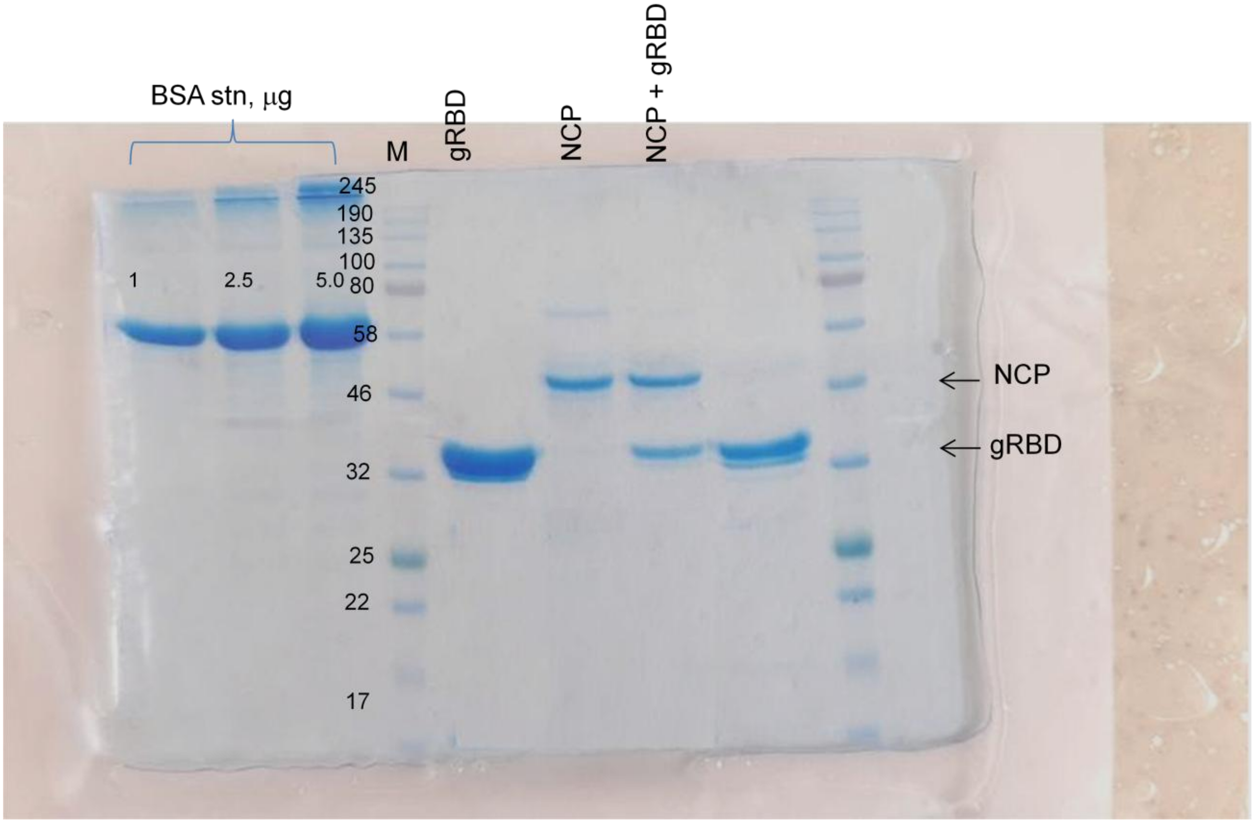

Supplementary Figure S8. SDS-PAGE analysis of RBD, N, and N+RBD proteins, eluted from a Sephacryl® S-200 HR column. Figure 3B was cropped from this SDS-PAGE gel image.

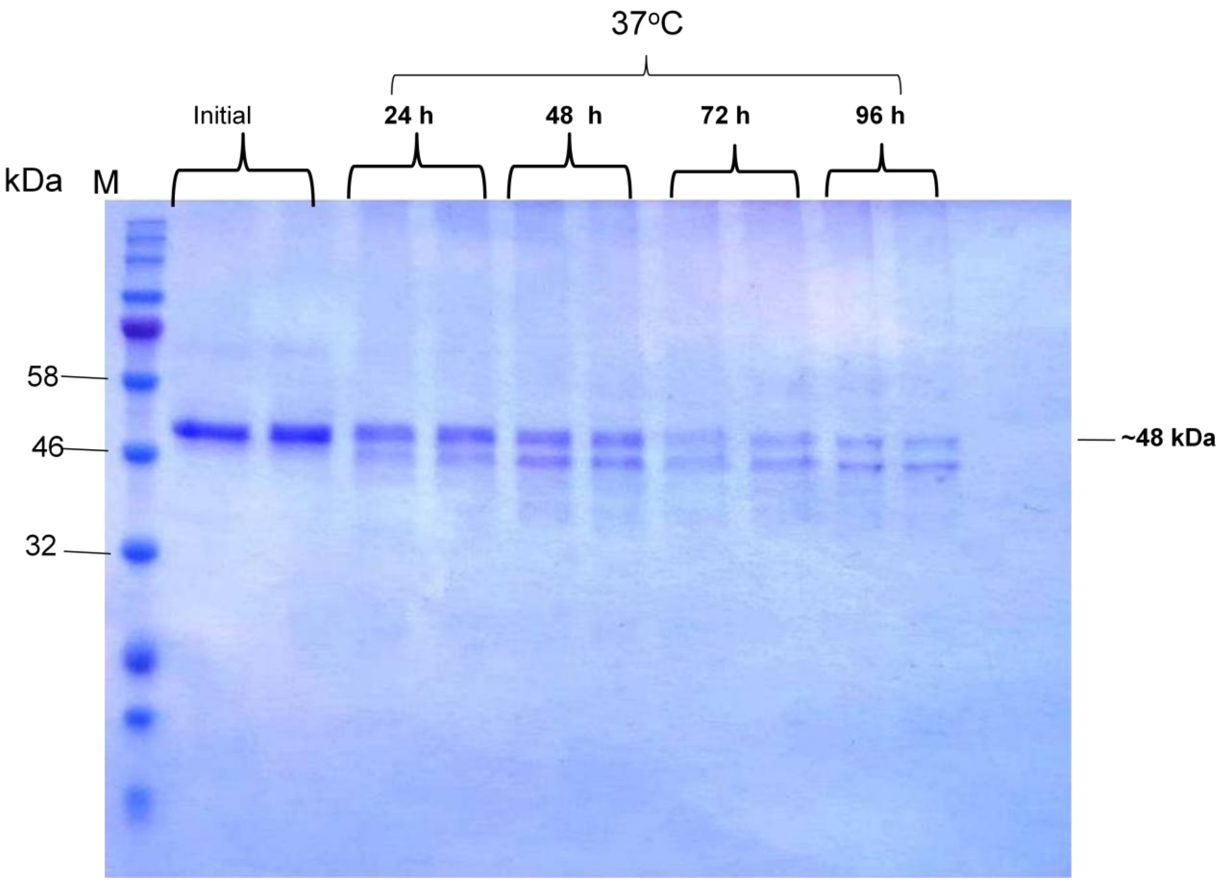

Supplementary Figure S9. Stability of plant-produced N protein. Plant-produced, FLAG antibody affinity column-purified N protein was incubated at 37 °C for 24, 48, 72, and 96 h, and analyzed in SDS-PAGE. Figure 4A was cropped from this SDS-PAGE gel mage.

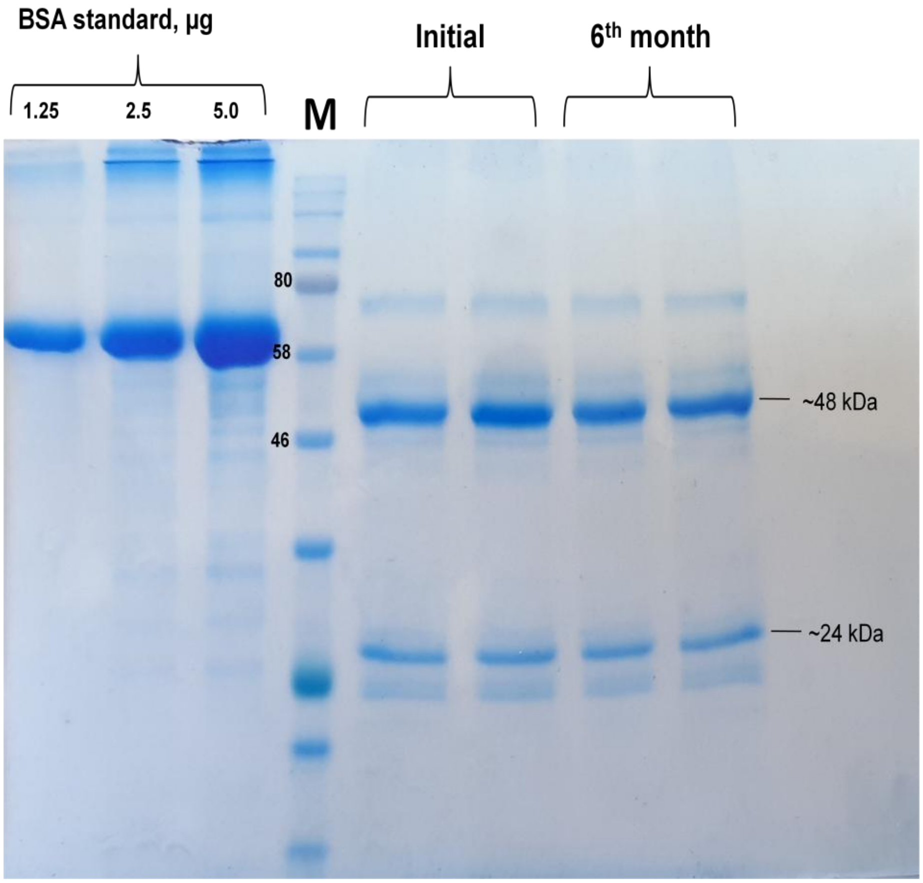

Supplementary Figure S10. Plant-produced, FLAG antibody affinity column-purified N protein was stored at −80°C for 6 months and then analyzed in SDS-PAGE M: color pre-stained protein standard. Figure 4B cropped from this SDS-PAGE gel mage.
